# Entrepreneurial homeworkers

**DOI:** 10.1007/s11187-020-00356-6

**Published:** 2020-05-18

**Authors:** Nam Kyoon N. Kim, Simon C. Parker

**Affiliations:** 1 Ivey Business School, Western University, London, Ontario Canada; 2 University of Aberdeen, Aberdeen, UK

**Keywords:** Homework, Self-employment, Entrepreneurship, Employers, Caregiving, J13, J24, L26, M13, R23

## Abstract

Nearly 40% of British self-employees are homeworkers. Using a large representative sample of the UK longitudinal survey data, we explore the determinants of self-employed homeworking, distinguishing between genders. We reject the notion that homeworking is a transitional entrepreneurial state that the self-employed “grow out of”, while establishing that both employer status and business structure play an important role in predicting which self-employed become homeworkers. Our findings also shed light on two outstanding puzzles in entrepreneurship scholarship: why so few of the self-employed create jobs for others, and why on average the self-employed suffer an earnings penalty compared with employees.

## Introduction

The regional entrepreneurship literature continues to grow rapidly, reflecting enduring interest in understanding entrepreneurs’ venture location choices and the resulting implications for regional economic development. Recent research has analyzed, inter alia: the determinants of persistent differences in regional rates of entrepreneurship (Andersson and Koster 2010; Bishop and Shilcof 2017; Fotopoulos and Storey 2017; Koster and Hans 2017; Modrego et al. 2017); regional firm entry and exit rates (Bishop and Shilcof 2017; Li 2017); the location and mobility of entrepreneurs (Di Addario and Vuri 2010; Kulchina 2016); regional knowledge spillovers as an entrepreneurship attractor (Audretsch et al. 2006; Bonaccorsi et al. 2014); and the concentration of entrepreneurship in neighborhoods, cities and clusters (Di Addario and Vuri 2010; Pe’er and Keil 2013). This valuable background has greatly enhanced our understanding of regional aspects of entrepreneurship.

What is much less well-known is why many entrepreneurs locate their ventures at their place of residence. It turns out that *entrepreneurial homeworkers* are surprisingly prevalent. For example, according to the present study, which utilizes data from the British Household Panel Survey (BHPS)—a representative longitudinal dataset of individuals and households residing in the UK—nearly 40% of the self-employed in the UK are homeworkers. This fact appears to be little known, in part because apart from a handful of prior studies (Mason et al. 2011; Reuschke 2016), entrepreneurial homeworking has been largely neglected as a research topic. This neglect is surprising for several reasons. First, homeworking influences entrepreneurs’ ability to serve lucrative markets and scale their businesses by hiring workers. Hence, it bears on entrepreneurial performance. Second, homeworking carries implications for understanding who participates in entrepreneurship. This is of interest to researchers concerned with the drivers of entrepreneurial selection. Third, the joint emergence of high-tech occupations and the decline of “traditional” employment provided by large employers make homeworking a topical alternative mode of employment. There is considerable policy interest in discovering more about the prevalence of homeworking in the new digital economy (Blount 2015; Huws et al. 2018).

Moreover, uncovering the determinants of entrepreneurial homeworking can inform several prominent areas of entrepreneurship research. These include how entrepreneurs manage work-life balance, where they locate their ventures, and how public policy influences entry into entrepreneurship. First, emerging research demonstrates that many people value the flexibility of being self-employed, and often “blur” home and work commitments to enhance their well-being and satisfaction (Boden 1999; Hurst and Pugsley 2011; Sevä et al. 2016). While much research has emphasized the importance of variations in work hours for achieving flexibility, the potential role of homeworking in this regard (whereby entrepreneurs can quickly switch between performing home and work duties) has been rather neglected. Second, entrepreneurship scholars are increasingly investigating entrepreneurs’ venture location choices (Dahl and Sorenson 2010; Koster and Venhorst 2014; Kulchina 2016; Stam 2007), and how these choices affect the performance of their ventures (Di Addario and Vuri 2010; Frederiksen et al. 2016). Yet this body of research is largely silent about homeworking, even though it may allow entrepreneurs to economize on commuting costs and rents, while facilitating flexible working. Third, public policy is known to favor entrepreneurship through, for example, various programs that promote self-employment as a career choice (Caliendo and Kritikos 2010; Michaelides and Benus 2012; Parker 2018, Chaps. 18–21). Policymakers also design policies to encourage entrepreneurs to create jobs for others (Henrekson and Johansson 2010; Mathur 2010). To date, however, few policy prescriptions have connected these hitherto disparate threads, even though (as we will argue) entrepreneurial homeworking may provide a basis for doing so.

Might entrepreneurial homeworking have been neglected because it is merely a transitional state, which entrepreneurs use temporarily before scaling up and professionalizing their ventures? The purpose of the present article is to investigate this possibility, and in the process to analyze the determinants of homeworking, including business structure and caregiving responsibilities. Using BHPS data, this paper uncovers salient determinants of selection into homeworking and provides evidence about whether homeworking is or is not a transitional phase for entrepreneurial ventures. As a byproduct, our findings also shed light on two outstanding puzzles in entrepreneurship scholarship, showing how homeworking can partly explain why so few of the self-employed create jobs for others, and why on average the self-employed suffer an earnings penalty compared with employees.

## Theory development

Our primary research question asks: “Is homeworking a transitional state, which entrepreneurs use to get started before scaling up and professionalizing their business?” In addressing this question, one needs to determine what factors influence entrepreneurs’ decision to work at home rather than in a separate, dedicated workplace. To that end, we draw on a broad body of literature relating to occupational choice models that analyze the decisions of individuals about whether to become entrepreneurs. The “Background literature” section will first briefly review background literature on entrepreneurship as an occupational choice, and key factors associated with entrepreneurial homeworking. The “Factors influencing homeworking” section then develops several hypotheses which apply occupational choice-based logic to the entrepreneurial homeworking setting: the goal is to identify factors that influence entrepreneurial homeworking. The “Is homeworking a transitional state?” section then explores factors associated with entrepreneurs using homeworking as a transitional state. The reason for this ordering of the conceptual discussion is that influencing factors need to be considered before the implications of transitioning out of being an entrepreneurial homeworker can be understood.

### Background literature

Occupational choice models of entrepreneurship analyze the decisions of individuals who have heterogeneous tastes and abilities and decide whether to become entrepreneurs or employees. Individuals derive utility from both financial factors like profits and non-financial factors such as autonomy (Taylor 1996) or job satisfaction (Guerra and Patuelli 2016). Individuals compare the utility they expect to derive from entrepreneurship with that obtainable from their next best alternative, which is usually taken to be wage employment (Parker 2018, Chap. 2).

Some occupational choice models focus only on utility derived from financial returns (e.g., Evans and Jovanovic 1989; Kihlstrom and Laffont 1979). They recognize that individuals choose not only whether to become an entrepreneur but also how many factors of production to utilize, e.g., capital and/or labor. For example, Kihlstrom and Laffont (1979) jointly analyzed the decision to become an entrepreneur with the choice of how many workers to employ, in a model where individuals differ from each other in terms of their risk tolerance. Entrepreneurship is widely recognized as a risky occupation: Kihlstrom and Laffont (1979) demonstrated that the most risk-tolerant people are both more likely to become entrepreneurs *and* to hire workers.

Other models introduce non-financial considerations into the mix. Evidence shows that non-financial factors influence entrepreneurs’ choices (Burke et al. 2000; Hurst and Pugsley 2011; Taylor 1996). These factors include a desire for creativity and autonomy (Block and Koellinger 2009; Hytti et al. 2013; Schneck 2014), and intrinsic satisfaction from work (Guerra and Patuelli 2016; Lange 2012). Another relevant factor is the flexibility afforded by self-employment. Entrepreneurs work longer hours on average than employees but often enjoy greater latitude over when they work and what tasks they perform there (Hyytinen and Ruuskanen 2007). Flexibility can be especially beneficial for entrepreneurs having household and caregiving responsibilities, who need to juggle their time between work and home (Boden 1999; Hurst and Pugsley 2011; Sevä et al. 2016). Overall, the evidence suggests that entrepreneurs care about both financial payoffs and non-financial factors when deciding whether to run a business (Block and Koellinger 2009). This insight informs our theorizing below, which incorporates both financial and non-financial components of utility into entrepreneurs’ decision-making calculus.

Other research has recognized that the occupational choice of entrepreneurship does not take place in a spatial vacuum. Several reasons explain why many entrepreneurs prefer to situate their ventures close to, or at, their homes. This may be where their customer base and other stakeholders are located (Stam 2007). The local nature of social networks and the need to reduce commuting costs given long work hours also explain why many entrepreneurs live close to where they work. Locating a venture far from home risks weakening the effectiveness of social capital. In addition, the entrepreneur may want to work close to dependents who need their care and support.

Relatively few studies to date have examined entrepreneurial homeworking. Two important exceptions are Mason et al. (2011) and Reuschke (2016). Mason et al. (2011) provide a descriptive analysis of home-based small- and medium-sized businesses in the UK, emphasizing the regional aspects of such businesses. Mason et al. (2011) dismiss as inaccurate the stereotype that home-based ventures are part-time, small and marginal. Instead, “the majority of home-based businesses are serious undertakings, occupying their own dedicated space, operating on a full-time basis, and based at home largely for business rather than for lifestyle reasons... These findings challenge the simplistic stereotype that dismisses home-based businesses as part-time, small and marginal and, therefore, of little economic significance” (Mason et al. 2011, p. 634). Mason et al. (2011) build on earlier work which looked exclusively at women home-based business owners (Jurik 1998; Loscocco and Smith-Hunter 2004).

Reuschke (2016) in contrast used the British Household Panel Survey (BHPS) data to explore how housing characteristics affect entries into self-employment separately for homeworkers and non-homeworkers. Reuschke (2016) proposed that housing influences differently entry into homeworking versus non-homeworking. She pointed out that housing provides not only financial benefits as a source of collateral to relieve borrowing constraints but also an affordable and versatile space in which to work flexibly—a precondition for many people such as those with caregiving responsibilities.

### Factors influencing homeworking

Below, we will focus on three major potential influences on entrepreneurial homeworking: employing others, forming business partnerships, and discharging caregiving responsibilities. We commence by noting that many residences are spatially constrained and lack the facilities to accommodate employees on-site (Reuschke 2016). This may prevent entrepreneurs from being able to offer a comfortable working environment to employees, inducing entrepreneurs to eschew homeworking in favor of using a dedicated office or factory location outside the home. In addition, many employees expect standard working conditions and hours, and access to standard office resources which may be lacking in somebody else’s home. For instance, unless a home is exceptionally commodious, and can be renovated to house regular office facilities and equipment, it may be difficult to offer employees the kinds of working conditions they are used to in larger firms.

In response, a home-based entrepreneur might be willing for employees to work remotely, e.g., at clients’ premises. But remoteness raises the risk of agency costs arising from moral hazard: it may be difficult for the entrepreneur to monitor and supervise workers and prevent them from slacking (Demsetz 1983; Jensen and Meckling 1976). One solution to this problem is for the entrepreneur to rent a central, dedicated office location, which is the logical alternative to homeworking. In such a location, the entrepreneur can work alongside their employees, making monitoring simpler and more effective. For all these reasons, one would therefore expect that, in the context of maximizing expected financial returns, employer entrepreneurs are less likely to be homeworkers:

- **Hypothesis 1** *The propensities of entrepreneurs to choose homeworking and hire employees are negatively related.*

Setting up a venture from home may entail another drawback too: it can be harder for entrepreneurs to work together with other business partners and operate more financially ambitious enterprises. In general, business partners tend not to be residentially co-located, and they are rarely selected on that basis. Non-co-located partners of a home-based business must therefore choose which partner’s home the business will be situated at. Home location is likely to impose monitoring costs on the business partner whose residence is not being used as the business location, as discussed above in the context of employees. While this is not necessarily an insuperable complication, it can entail time-consuming and ongoing negotiations between business partners, reducing the attractiveness of home-based venturing for one or more of the business partners. For these reasons, one might expect homeworking to be better suited to sole proprietorships than to business partnerships.

There is also likely to be a gender dimension to this issue. Evidence shows that a smaller proportion of women than men incorporate their businesses using legal forms like business partnerships relative to sole proprietorships (Brush 1992; Klapper and Parker 2010). There are many reasons for this, reflecting in part the pronounced concentration of women in businesses involving part-time work and located in industry sectors with limited opportunities for growth (Parker 2018, Chap. 8). Usually, only larger-scale ventures can justify and support multiple business partners, and these tend to be operated more by men than women. We summarize these arguments as follows:

- **Hypothesis 2a** *The propensities of entrepreneurs to choose homeworking and form their businesses as partnerships are negatively related.*
- **Hypothesis 2b** *The negative relationship between homeworking and business partnerships is stronger for men than for women entrepreneurs.*

Another factor that bears on the homeworking choice relates to caregiving responsibilities. If externally provided childcare or eldercare is costly, homeworking might be attractive to entrepreneurs by offering them a flexible way to combine work and domestically provided caregiving (Hyytinen and Ruuskanen 2007). Many entrepreneurs have pressing commitments relating to the home, including caregiving responsibilities which can make working away from home problematic or onerous (Craig et al. 2012; Edwards and Field-Hendrey 2002). For instance, evidence shows that household members—especially women—who look after disabled people, or who have young children, are more likely to be self-employed (Craig et al. 2012; Rønsen 2014; Wellington 2001).

Homeworking economizes on commuting costs, which is relevant to caregivers who must attend to the recurring needs of others, especially in cases where the caregiver has to devote regular periods of personal care. Since children and disabled relatives are often located in or near the caregiver’s home (Sit et al. 2004), homeworking can reduce these costs. Given the well-known flexibility of self-employment as a way of juggling work and life responsibilities (Gimenez-Nadal et al. 2012; Thébaud 2015), these arguments suggest that entrepreneurs with caregiving responsibilities find homeworking an especially attractive option to maximize the flexibility of entrepreneurship.

In the literature to date, the potential for homeworking to help entrepreneurs juggle business and caregiving responsibilities has been explored principally for women entrepreneurs specifically, since women devote more time on average to caregiving responsibilities than men do (Craig et al. 2012; Edwards and Field-Hendrey 2002). According to Carr (1996), 20% of self-employed women worked from home, compared with just 6% of self-employed men. Craig et al. (2012) analyzed Australian Time Use Survey data and estimated that working from home is highly correlated with self-employment among mothers, who used self-employment to combine earnings and childcare, whereas fathers prioritized paid employment.

It is important at this juncture to distinguish between two different loci of choice for entrepreneurs who want to combine business and caregiving activities. One locus of choice is between entrepreneurship versus paid employment—the occupational choice discussed above. The other is between homeworking and non-homeworking *conditional on being an entrepreneur*. It is possible that homeworking provides the most convenient way of juggling business and caregiving duties. However, it is also possible that self-employment provides entrepreneurs with enough flexibility that they can discharge their caregiving duties without having to locate their venture at home as well. Nevertheless, we state the following hypotheses:

- **Hypothesis 3a** *The propensity of entrepreneurs to choose homeworking is positively associated with their caregiving responsibilities.*
- **Hypothesis 3b** *The positive relationship between homeworking and caregiving is stronger for women than for men entrepreneurs.*

### Is homeworking a transitional state?

Below, we discuss how hiring employees and forming business partnerships might only occur once the entrepreneur has ceased homeworking, and works from a separate, dedicated, location. Throughout, the theoretical discussion draws on the literature introduced in the preceding subsections, extending its logic to the question at hand.

As noted in the “Background literature” section, entrepreneurship is risky. In response, entrants may craft risk-minimization strategies. According to Folta et al. (2010), “hybrid” entrepreneurship is one such strategy, whereby employees continue working in their main job in paid employment while “dipping a toe” in the entrepreneurial waters to see if it is worth making a full-time transition. Real options logic (Dixit et al. 1994) suggests that entrants seek to avoid heavy early-stage investments until they are sure that the payoff in entrepreneurship is high enough to merit them. One such costly investment is in arranging to secure and equip an office location for a new venture, since if the venture fails to thrive, the associated costs are largely sunk and unrecoverable. In contrast, homeworking is cheap and involves little cost in setting up. Real options logic then predicts that only if hybrid entrepreneurs generate sufficiently high returns will they choose to go full-time. At that point, it makes sense to sink investments, including arranging office facilities.

In contrast, this logic does not apply to non-hybrid entrants who switch completely into full-time entrepreneurship. These entrants may have different motives: e.g., they may have quit their job; are risk-neutral or risk-loving; prefer to work full-time on their venture; or start non-capital-intensive firms. Such people may still benefit from homeworking, as discussed below; but the motive of choosing homeworking as a risk-minimization strategy would be weak or absent. Thus, if homeworking is a temporary, transitional arrangement, we would expect to see relatively high annual inflows of hybrid entrepreneurs into homeworking.

In terms of outflows, if homeworking is a temporary solution for early-stage ventures, some of these ventures should shift to become non-homeworking businesses as they scale and professionalize. Scaling often entails hiring employees (Delmar et al. 2003)—so, for the reasons given in the lead up to Hypothesis 1, a home-based location may become unsuitable for new employees. That may require home-based solo entrepreneurs to quit homeworking and move the business into a conventional workspace instead. Similar arguments apply to business partnerships: one might expect entrepreneurs who are professionalizing their ventures and taking on additional managerial and investment capacity embodied in another partner to replace homeworking with non-homeworking to facilitate that transition (see Hypothesis 2). Summarizing these predictions, we have:

- **Hypothesis 4a** *Homeworking status in entrepreneurship is positively associated with transitions into non-homeworking employer status in entrepreneurship the following year.*
- **Hypothesis 4b** *Homeworking status in entrepreneurship is positively associated with transitions into non-homeworking business partnership status in entrepreneurship the following year.*

## Data and methods

### The dataset

Data come from the British Household Panel Survey (BHPS), an annual, nationally representative UK-wide household survey for social and economic research first administered in 1991 (Buck et al. 2006). The first cohort consisted of 10,300 individuals across Great Britain. Since 2001, respondents have been drawn from across the whole of the UK, i.e., Great Britain plus Northern Ireland. Members of the original households were followed up with many participants agreeing to be re-interviewed in subsequent years. The BHPS is well-suited to analyzing homeworkers as it contains rich data on the self-employed, homeworking, and other salient variables discussed above.

For most of the analysis that follows, we draw panel data from 2004 to 2008, the last 5 years the BHPS was conducted. The sample frame in this study is all members of the Great Britain (England, Scotland, and Wales) workforce aged 16 years of age and over, who are either employed or self-employed. Observations from Northern Ireland were excluded owing to data limitations. Individuals who declared themselves retired, the long-term sick and disabled, and students were also excluded from the sample. Although data on employees will be used to provide some subsequent checks on the results, the main results will focus only on the self-employed sample and the homeworking subsample. No upper age limit on the self-employed was imposed, as a non-trivial proportion (5.5%) of our self-employed sample are aged 65 and over. However, while subcontractors are coded as self-employed individuals in BHPS dataset, they are closer to employees on contract. Therefore, following Reuschke (2016), we removed subcontractors from our sample.

### Variable definitions

#### Dependent variable

##### Entrepreneurial homeworkers

Prior researchers have measured homeworking in the entrepreneurial context as cases “where the work (production or service) occurs in the home, and those where the work occurs away from the home with the home serving as the administrative base... [The entrepreneurs are engaged in] selling products or services into the market operated by a self-employed person, with or without employees, that uses residential property as a base from which the operation is run” (Mason et al. 2011, p. 629). Consistent with this definition, “those self-employed workers who work at home or from their own home are considered as home-based businesses” (Reuschke 2016, p. 384).

In the following, we will define a homeworker entrepreneur, *Homeworker*, in precisely the same way as just described. Indeed, the BHPS codebook defines self-employed homeworkers as “those who work principally at or from home.” The other possible work locations are working from separate business premises; working from customer or client premises; or working at some other place such as a van or a stall. These are all non-homeworkers. For example, a self-employed accountant could choose to sell their services from home using a phone and personal computer: they would be classified as a homeworker. Alternatively, the same accountant may prefer to rent office space close to their clients, interacting with them there: they would not be classified as a homeworker.

While self-employment is a widely used measure of entrepreneurship (Parker 2018, Sec. 1.3), it is not without its limitations. Self-employment can encompass individuals who are unlikely to be entrepreneurs by other criteria, such as innovation or novelty. It also omits many nascent entrepreneurs, who have not yet changed their labor market status to self-employed (Parker and Belghitar 2006). On the other hand, almost all self-employment involves the entrepreneurial functions of risk-bearing and business ownership and control. Self-employment is a prominent and important segment of the labor market, accounting for over 10% of the workforce in the UK, the USA, and many other countries (Parker 2018, Chap. 1). Many self-employed are business owners whose activities have been linked to aggregate productivity, wealth creation, job generation, innovation, and growth (Guiso and Schivardi 2011; Koellinger and Thurik 2012). Since *all* entrepreneurs own their own business and do not have an employer, the self-employment measure possesses the merit of inclusiveness. Another advantage is that self-employment data are also widely available in household surveys, which facilitates the analysis of homeworking.

There are 4696 self-employed observations in our BHPS sample, yielding a self-employment rate of 12.4%. Of these, 36.9% (i.e., 1734 cases) are homeworkers. This fraction exceeded 40% in the final wave of the BHPS (2008). In contrast, less than 2% of employees in the BHPS sample work from home. Employee homeworkers are not the central focus of this paper; some descriptive analysis later demonstrates that they are not comparable to self-employed homeworkers in several important respects and probably merit a separate empirical (as well as theoretical) treatment.

#### Independent and control variables

The main independent variables operationalize the constructs introduced in Hypotheses 1–3. They are measured as follows. First, the variable *Employer* is coded as 1 for self-employed individuals who employ others, and 0 for self-employed individuals who work alone. Second, we code a dummy variable *Business partnership* for whether the respondent has one or more business partners in terms of the legal ownership of business. Third, we measured caregiving with two dummy variables—*Caregiver* (*disabled*), and *Caregiver* (*child*). *Caregiver* (*disabled*) equals 1 if the respondent looks after a disabled person, and 0 otherwise. Likewise, *Caregiver* (*child*) equals 1 if the respondent looks after a child under age 16, and 0 otherwise. Fourth, since some of the hypotheses distinguish between males and females, we coded the variable *Female* to equal one if the respondent is female and zero if they are male. Finally, we measured *Hybrid entrepreneur* with a dummy variable which equals 1 if the respondent’s main job is a paid employment with a self-employment second job, and 0 if the respondent is self-employed but does not have a second job. This follows the definition of hybrid entrepreneur by Folta et al. (2010), which is defined as “individuals who engage in self-employment activity while simultaneously holding a primary job in wage work” (p 254).

We included numerous control variables in our empirical analysis to reduce the risk of omitted variable bias. We briefly outline these together with a rationale for their inclusion. First, it has been suggested that technological change has made operating home-based businesses more feasible than it used to be. Entrepreneurs who work in high-tech occupations (e.g., R&D or legal services) may find it easier to connect remotely with customers and suppliers and so operate their business effectively from home. We coded a variable *High-tech occupation* as a binary variable using the BHPS SOC 2000 classification. Science, technology, research, design, legal, and financial professions are coded as high-tech occupations. Table 7 in the Appendix provides a detailed list.

Second, demographic variables may also affect homeworking. For example, married entrepreneurs may be more financially secure and so better able to support flexible homeworking that can accommodate other household responsibilities such as caregiving (Werbel and Danes 2010). We accordingly coded a dummy variable called *Married.* In addition, abundant research has linked human capital with self-employment, which may affect various types of self-employment differently (Parker 2018, Sec. 5.2). We therefore included two human capital controls: a dummy for whether the respondent is a graduate, *Graduate*; and age as a broad measure of experience, *Age*. And, since homeworking may be the only way that individuals in poor health can operate a business, we also added a control variable *Health limits work.*

Third, social capital may be linked to homeworking to the extent that a home-based business leverages local social networks and resources and so overcomes locational disadvantage. A control variable *Social capital* is implemented as a multi-item measure, with three self-reported items from the BHPS, all on five-point Likert scales, as follows. Respondents rated the degree to which they: belong to their neighborhood; can access advice locally; and talk to neighbors. These items all correspond to well-established notions of social capital implemented in prior research (e.g., Lochner et al. 1999). Cronbach’s alpha for these items is 0.74, suggesting good internal consistency.

Fourth, individual preferences may also influence choices. For example, if homeworking offers a safer environment, removing exposure to expensive business premise leases, more risk-averse people might become homeworkers. To measure risk tolerance, we used the following BHPS question: “Are you generally a person who is fully prepared to take risks or do you try to avoid taking risks?”. Respondents answered on a 10-point scale, ranging from 1 (“won’t take risks”) to 10 (“ready to take risks”). This serves as a direct, self-reported measure called *Risk tolerance.* Survey measures of risk tolerance are widely used by economists, and they appear to be reliable (Dohmen et al. 2012).

Fifth, freelancing arguably lends itself more readily to homeworking than other self-employed individuals, since freelancers often enjoy freedom about where they work. A dummy variable *Freelance* is therefore included as a control. We also added five industry dummy variables that are salient for homeworkers. *Retail*, *Construction*, *Education*, *Health and social work*, *Recreational*, *cultural and sporting activities* are coded as one if individual is working in respective industries, and zero otherwise. Tables 8, 9, 10, 11, and 12 of the Appendix outline the industry sectors that are included as dummy variables and the number of homeworkers in those industries. Additionally, a dummy variable *Full-time venture* is included which equals one if the self-employed is working full-time for their business, and zero otherwise. We also created a dummy variable *Switch* based on current and previous employment status, coded as one if self-employed respondents had switched into their current business from paid employment, and zero otherwise.

Sixth, previous research (e.g., Reuschke 2016) has identified housing characteristics as possible determinants of homeworking decisions. Thus, we control for the *Number of rooms*, since more rooms presumably makes it easier for the entrepreneur to set up a home office. We also use dummy variables to control for the type of dwelling, such as *Detached*, *Semi-detached*, *Terraced*, and *Flat* (i.e., apartment). It also seems plausible that homeowners face fewer restrictions than renters on configuring their dwellings for business use. Hence, we added the dummy variable *Homeowner* to the list as well.

Seventh, regional characteristics may also affect the homeworking decision. To start with, regions with high average house prices tend also to have higher business office rents (Dobson and Goddard 1992) and so are more likely to induce entrepreneurs to work from home if they can. In addition, higher regional average earnings tend to be concentrated in urban centers where there is a higher density of consumers (Dumais et al. 2002). The greater that demand, the more profitable it will be for entrepreneurs to locate their businesses nearby, to maximize their ability to capture the value associated with it. This may reduce homeworking, as may a non-urban location for the entrepreneur’s main residence.

To control for these possible factors, we obtained confidential local authority districts (LAD) data from the BHPS and matched it to the individual-level dataset. LADs are a level of subnational division of the UK used for the purposes of the local government. There are 326 LADs in England, 22 in Wales, and 32 in Scotland. LAD-level average house price data from 2004 to 2008 were obtained from the UK House Price Index (UK HPI) dataset. The UK HPI uses house sales data from HM Land Registry, and Registers of Scotland. House sales data are compiled by the Office for National Statistics (ONS). We used average house price data of each LAD as of December of each calendar year and log-transformed the numbers to generate the control variable *House price*. Average earnings for each LAD were taken from ONS, *Earnings and hours worked*, *place of residence by local authority*: *ASHE* Table 8*.* We used Table 8.7a (annual pay) of each year during 2004–2008. We had to map each ASHE unit code to corresponding LAD codes to transform the data to LAD level. These data were used to compute a log-transformed *Local earnings* variable. Note that for all cases, *House price* and *Local earnings* exhibit variation from year to year. Additionally, we collected data on the local unemployment rate, *Unemployment rate*, at the LAD level from UK’s Office for National Statistics (ONS). Finally, we also collected data on urban/rural classifications from the Government of the UK (for England), Scottish Government (for Scotland), and ONS (for Wales) to code a dummy variable *Urban*, which equals one if the respondent lives in an urban and zero if they live in a rural area. Details about these data sources can be found in Appendix 2.

### Descriptive statistics

Table 1 provides some descriptive statistics about the characteristics of self-employed homeworkers (“H/w”), vis-a-vis non-H/w self-employed and employees. Table 2 presents the pairwise correlation matrix. We start by comparing self-employed homeworkers with self-employed non-homeworkers in columns (1) and (2). Some comparisons with employees in general (columns (4) and (5)) will also be made.

**Table 1:** Descriptive statistics

	Self-employed		Self-employed # of obs (3)	Employee		Stat. test (1) vs (2) (6)
	Hw (1)	Non-Hw (2)		Hw (4)	Non-Hw (5)	
Homeworking, %	36.9	63.1	4696	1.6	98.4	
Employer, %	14.8	32.9	4553	2.4	4.5	*χ*^2^(1) = 210.5***
Business partnership, %	18.3	26.2	3490			*χ*^2^(1) = 39.8***
Caregiver (disabled), %	5.4	3.8	3757	24.8	19.5	*χ*^2^(1) = 4.7*
Caregiver (child)	18.4	11.2	4696	25.8	19.9	*χ*^2^(1) = 44.3***
Female, %	34.8	25.3	4488	52.6	52.2	*χ*^2^(1) = 35.2***
Hi-tech occupation, %	17.3	11.8	4696	14.9	10.4	*χ*^2^(1) = 26.6***
Married, %	82.1	79.1	4696	88.2	71.2	*χ*^2^(1) = 4.0*
Graduate, %	18.3	19.7	4169	34.6	20.2	*χ*^2^(1) = 2.0
Age: mean	48.8	44.6	4696	45.7	39.6	F (1, 4694) = 102.7***
(St. dev.)	(12.2)	(12.0)		(12.0)	(12.6)	
Health limits work, %	10.4	8.5	4556	7.8	7.4	*χ*^2^(1) = 4.2*
Social capital: mean	0.8	0.6	3182	0.7	0.6	F (1, 3180) = 43.1***
(St. dev.)	(0.7)	(0.7)		(0.8)	(0.8)	
Risk tolerance: mean	6.2	6.3	3141	6.2	5.8	F (1, 3139) = 3.0 †
(St. dev.)	(1.9)	(1.9)		(1.9)	(1.9)	
Freelance, %	7.6	6.3	4484			*χ*^2^(1) = 0.9
Retail sector, %	5.2	10.0	4696	8.0	14.0	*χ*^2^(1) = 38.3***
Construction, %	14.8	22.7	4696	6.8	5.2	*χ*^2^(1) = 29.4***
Education, %	5.8	3.5	4696	6.6	10.3	*χ*^2^(1) = 12.8***
Health and social work, %	8.0	5.7	4696	6.6	14.0	*χ*^2^(1) = 6.4*
Recreational, %	5.6	5.3	4696	2.5	2.3	*χ*^2^(1) = 0.0
Full-time venture, %	34.5	39.2	4484			*χ*^2^(1) = 26.0***
Switch from employee, %	6.2	8.2	4696			*χ*^2^(1) = 3.5†
Number of rooms: mean	5.7	5.3	4583	6.1	4.8	F (1, 4222) = 52.0***
(St. dev.)	(2.3)	(1.9)		(2.1)	(1.6)	
Detached house, %	39.1	31.1	4696	53.8	22.8	*χ*^2^(1) = 26.2***
Semi-detached house, %	25.7	27.6	4696	24.9	33.9	*χ*^2^(1) = 2.3
Terraced house, %	17.8	20.6	4696	12.2	25.9	*χ*^2^(1) = 2.8†
Flat house, %	6.8	10	4696	4.9	11.3	*χ*^2^(1) = 15.0***
Homeowner, %	86.8	85.5	4609	88.2	80.9	*χ*^2^(1) = 0.1
Log local house price: mean	12.0	12.0	4696	12.0	12.0	
(St. dev.)	(0.3)	(0.3)		(0.3)	(0.3)	
Log local earnings: mean	10.0	10.0	4525	10.0	10.0	
(St. dev.)	(0.2)	(0.1)		(0.1)	(0.1)	
Local unemployment	4.9	5.0	4584	4.7	5.1	
(St. dev)	(1.9)	(1.9)		(1.9)	(1.9)	
Urban, %	48.7	57.1	4696	56.5	65.1	*χ*^2^(1) = 30.3***
Max. no. obs.	1734	2962	4696	515	31,763	

*p < 0.05

**p < 0.01

***p < 0.001 (two-tailed test)

**Table 2:**
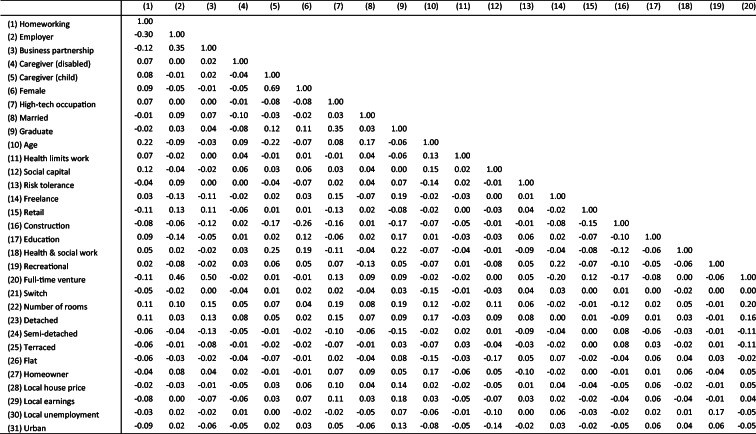

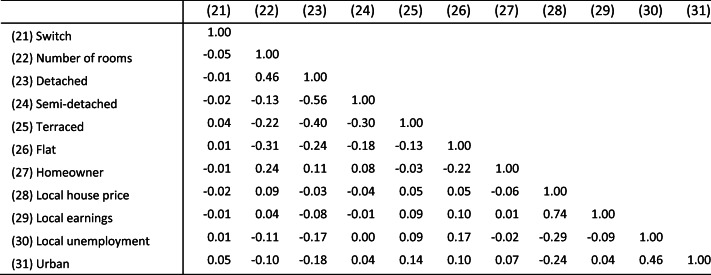
Correlation matrix

Self-employed homeworkers are substantially and significantly (*p* < 0.001 using a χ^2^ test) less likely to be employers than other self-employed. This proffers some preliminary support to Hypothesis 1. Second, self-employed homeworkers are also significantly less likely (*p* < 0.001 using a χ^2^ test) than other self-employed to be in a business partnership, but are significantly more likely to be caregivers of disabled family members (*p* < 0.05 using a χ^2^ test) and dependent child (*p* < 0.001 using a χ^2^ test)—in accordance with Hypotheses 2a and 3a.

Turning to the control variables, there is no significant difference between homeworkers and other self-employed in terms of home ownership (*p* > 0.5). Hence, homeworking is not clearly associated with home-owning status. The same is true of being a graduate. Among the self-employed, women are disproportionately found in the homeworker category (*p* < 0.001), as would be expected if women entrepreneurs value flexibility more than men. This difference within the self-employed group also applies to work-limiting health conditions (*p* < 0.05). Among the self-employed, homeworkers are significantly less likely (*p* < 0.001) to be in the retail or construction sectors, as expected. On the other hand, homeworkers are significantly more likely to be in the education (*p* < 0.001) and health and social work sectors (*p* < 0.05), compared with other self-employed individuals.

Self-employed homeworkers are also older on average than other self-employed workers. This difference is significant across the four groups in Table 1 according to ANOVA tests [F(1, 4694) = 102.7, *p* < 0.001]. Table 1 also suggests that self-employed homeworkers possess the most social capital. This difference is statistically significant across all four groups [F(3, 28,537) = 27.97, *p* < 0.001] as well as across the two self-employed groups [F(1,3180) = 43.1, *p* < 0.001]. Self-employed homeworkers are no more or less likely to be freelancers (*p* > 0.05), but they are less likely to live in urban areas than other self-employed workers (*p* < 0.001 using a χ^2^ test). Self-employed homeworkers are also less likely to be operating full-time (*p* < 0.001) but are more likely to be living in detached houses (*p* < 0.001) with more rooms [F(1, 4222) = 52.0, *p* < 0.001] than other self-employed workers.

Finally, Table 1 also indicates that self-employed homeworkers differ in several important respects from employee homeworkers. Employee homeworkers are significantly more likely than their self-employed counterparts to be female and caregivers, and to be graduates; they are also younger. Overall, these findings validate the choice to study self-employed homeworkers separately from employee homeworkers.

### Empirical methods

We test our hypotheses using a random effects probit model. A random effects model was preferred to a fixed effects model because it can estimate the effects of time-invariant variables. Moreover, a fixed effects model would only identify switchers into and out of homeworking, which drops too many observations and besides is not the sample of interest in this study.

It is possible that the variable *Employer* is endogenous. To explore this possibility, we used two different approaches. First, we estimated a Three-Stage-Least-Squares (3SLS) linear probability model, using type 1 (*Risk tolerance* for *Employer* equation) and type 2 instruments (*Caregiving* for *Homeworking* equation) for these two equations. These estimates (details available on request) revealed that homeworking is an insignificant predictor of being an employer. We also estimated a Seemingly Unrelated (SUR) bivariate probit model with two endogenous variables: *Homeworking* and *Employer*. A SUR bivariate probit model allows the error terms of two equations to be correlated. If the equations are not independent of each other, they must be estimated as a system. However, for this model, the Wald test of independent errors cannot be rejected, again suggesting an absence of endogeneity between the *Homeworking* and *Employer* equations (details also available on request). Based on these results, we proceeded to estimate *Homeworking* in a single equation specification.

Finally, to explore the possibility that homeworking is merely a transitional work arrangement that precedes larger-scale entrepreneurship, we also compute transition matrices and estimate probit models predicting transitions into homeworking and non-homeworking.

## Results

We first present the results from estimating the random effects probit model using the total sample, before presenting the results separately for males and females, consistent with our conceptual analysis. We then go on to explore whether homeworking is a transitory state for businesses.

Model (1) of Table 3 displays the estimation results for the total sample, while Models (2) and (3) do so for the male and female subsamples respectively. We see that the propensity of entrepreneurs to choose homeworking and hire employees is negatively related for the total sample (*p* < 0.001), the male subsample (*p* < 0.001), and female subsample (*p* < 0.05). Thus, Hypothesis 1 is supported. Also, business partnerships are negatively related to homeworking for the total sample (*p* < 0.01) and the male subsample (*p* < 0.001), supporting Hypothesis 2a. This relationship is weaker for females, consistent with Hypothesis 2b.

**Table 3:** Random effects probit models

	Random effects probit estimation		
DV = homeworker	(1) Total sample	(2) Male sample	(3) Female sample
Employer	− 1.41***	− 1.13***	− 3.67*
	(0.24)	(0.25)	(1.71)
Business partnership	− 0.61**	− 0.72*	− 0.30
	(0.21)	(0.23)	(0.66)
Caregiver (disabled)	0.09	0.40	− 1.35
	(0.39)	(0.45)	(1.00)
Caregiver (child)	0.89*	− 0.01	1.47*
	(0.36)	(0.64)	(0.71)
Female	0.20	Omitted	Omitted
	(0.35)		
Hi-tech occupation	0.36	0.19	1.44
	(0.28)	(0.28)	(1.43)
Married	0.20	− 0.13	1.93
	(0.35)	(0.36)	(2.41)
Graduate	− 0.70*	− 0.34	− 1.46
	(0.33)	(0.38)	(1.37)
Age	0.06***	0.06***	0.12†
	(0.01)	(0.01)	(0.07)
Health limits work	0.20	0.08	1.22
	(0.27)	(0.30)	(1.01)
Social capital	0.38*	0.38*	0.40
	(0.16)	(0.18)	(0.69)
Risk tolerance	0.00	0.04	− 0.15
	(0.06)	(0.06)	(0.30)
Freelance	0.31	0.35	− 0.35
	(0.30)	(0.37)	(0.63)
Retail	− 0.52	− 0.51	− 0.96
	(0.38)	(0.44)	(1.33)
Construction	− 0.12	− 0.09	0.43
	(0.27)	(0.26)	(4.21)
Education	1.24*	2.01***	0.31
	(0.60)	(0.62)	(1.27)
Health and social work	0.38	− 0.56	2.15
	(0.41)	(0.49)	(1.41)
Recreational	0.35	0.74	− 1.48
	(0.48)	(0.57)	(1.53)
Full-time venture	0.32†	0.35†	0.32
	(0.16)	(0.19)	(0.39)
Switch	0.16	0.04	1.35
	(0.35)	(0.41)	(1.09)
Number of rooms	0.14**	0.03	0.53***
	(0.05)	(0.06)	(0.16)
Detached	− 0.63	− 0.72	− 1.75†
	(0.55)	(0.74)	(0.90)
Semi-detached	− 0.99†	− 1.26†	− 1.50
	(0.54)	(0.72)	(0.99)
Terraced	− 0.82	− 1.01	− 1.12
	(0.56)	(0.75)	(1.12)
Flat	− 0.74	− 0.87	− 1.60
	(0.63)	(0.82)	(1.53)
Homeowner	− 0.37	− 0.23	− 1.28
	(0.31)	(0.35)	(0.82)
Local house Price	0.43	0.49	0.05
	(0.54)	(0.60)	(1.77)
Local earnings	− 1.54†	− 1.49	− 1.54
	(0.79)	(0.92)	(2.74)
Local unemployment	0.03	0.04	0.08
	(0.05)	(0.05)	(0.11)
Urban	− 0.22	− 0.25	− 0.58
	(0.23)	(0.26)	(0.86)
Number of observation	2177	1595	582
Log pseudolikelihood	− 889.23	− 661.90	− 201.27
Wald *χ*^2^	126.10	105.16	21.25

†*p* < 0.10

*p < 0.05

**p < 0.01

***p < 0.001 (two-tailed test)

Hypothesis 3a predicted that caregiving responsibilities are positively related to the propensity of entrepreneurs to choose homeworking. Also, Hypothesis 3b predicted that caregiving responsibilities will make self-employed women more likely to choose homeworking than self-employed men. Both Hypotheses 3a and 3b are supported when caregiving responsibilities entail having a dependent child in the household: the effect is statistically significant for females (*p* < 0.05) but not males. In contrast, having caregiving responsibilities for disabled persons is not significantly related to the homeworking decision of self-employed individuals. It is possible that self-employment provides enough flexibility to discharge caregiving responsibilities of a disabled person without the need to work from home.

Turning to the control variables, we find positive associations between homeworking and each of age, social capital, and working in the education sector for the total sample and the male subsample. The number of rooms in one’s house is also positively related to the homeworking decision, especially for women. Strikingly, though, few other relationships approach conventional levels of statistical significance—including having a work-limiting health condition. On this last point, it is again possible that self-employment provides sufficient flexibility to accommodate this without needing homeworking.

All of the results so far relate to influences on homeworking, as discussed in the “Factors influencing homeworking” section. We turn next to the primary research question, which is whether homeworking is a transitory state for fledgling businesses. As noted in the “Is homeworking a transitional state?” section, if that is the case, one might expect numerous hybrid entrepreneurs to transition into homeworking from year to year. To check whether this notion gains empirical support, Table 4 presents the year-to-year transition matrix for hybrid and non-hybrid entrepreneurs, calculated by pooling every wave of the sample. The table shows that, among non-hybrid entrepreneurs, there are relatively high annual transition rates (11–17%) between homeworking and non-homeworking. In contrast, virtually no hybrid entrepreneurs make transitions into homeworking—or even out of hybrid status. Hence, homeworking does not seem to be a risk-minimization strategy for hybrid entrepreneurs, casting doubt on the notion that homeworking is an entry point for entrants using this strategy. As a side note, the low rate of transitions out of hybrid entrepreneurship and into full-time entrepreneurship (1.8%) is striking, and contrast with the higher rate (8.5%) observed by Folta et al. (2010) in the Swedish context. It is not clear what explains this result. While it is of secondary interest in the present study, it nevertheless calls for further explication in future research.

**Table 4:** Transition matrix for hybrid and non-hybrid entrepreneurs

Group (vertical: previous, horizontal: current)	Not hybrid, non-homeworking	Not hybrid, homeworking	Hybrid entrepreneur	Total
Not hybrid, non-homeworking	1478	184	1	1663
(%)	(88.9)	(11.0)	(0.1)	
Not hybrid, homeworking	192	928	1	1121
(%)	(17.1)	(82.8)	(0.1)	
Hybrid entrepreneur	1	4	268	273
(%)	(0.4)	(1.4)	(98.2)	
Total	1671	1116	270	3057

Continuing with the question of whether homeworking is a transitory haven for fledgling businesses, we next tested Hypotheses 4a and 4b. To that end, we estimated a probit model with two dependent variables—(1) non-homeworking self-employed who are employing others, and (2) Non-homeworking self-employed with business partners. In accordance with the phrasing of Hypotheses 4a and 4b, last year’s homeworking status was included as the main independent variable.

Table 5 presents the results. We start with model (4), which reveals that the previous year’s homeworking status is positively related to the homeworking status in the current year (*p* < 0.001). Thus, self-employed homeworkers are significantly more likely to remain in this state than to transition to non-homeworking status. Strikingly, model (5) indicates that the previous year’s homeworking status without employing others is *negatively* related to current non-homeworking status while employing others (*p* < 0.001). Hence, we reject Hypothesis 4a, and infer that homeworking is not merely a temporary repository of businesses that subsequently hire employees and transition to non-home-based locations. Along similar lines, model (6) indicates that the previous year’s homeworking status without business partners is *negatively* related to current non-homeworking status with business partners (*p* < 0.001). This leads us to reject Hypothesis 4b. Overall, these results indicate that homeworking is more than a transitional phase for fledgling businesses.

**Table 5:** Probit regression models for transitioning

DV	(4) Homeworking status (current year)	(5) Non-homeworking and employing others	(6) Non-homeworking with business partners
Homeworking (previous year)	2.19***		
	(0.09)		
Homeworking not employing others (previous year)		− 3.58***	
		(0.31)	
Homeworking without bus partners (previous year).			− 3.19***
			(0.33)
Employer	− 0.48***		1.06***
	(0.11)		(0.20)
Business partner	− 0.25*	0.69***	
	(0.10)	(0.18)	
Caregiver (disabled)	0.13	− 0.44	0.23
	(0.18)	(0.35)	(0.31)
Caregiver (child)	0.29†	− 0.11	0.18
	(0.15)	(0.32)	(0.33)
Female	0.04	0.06	− 0.36
	(0.12)	(0.27)	(0.15)
Hi-tech occupation	0.02	− 0.57*	− 0.43
	(0.14)	(0.28)	(0.27)
Married	0.08	0.49†	0.82*
	(0.18)	(0.27)	(0.35)
Graduate	− 0.28*	0.56*	0.25
	(0.13)	(0.25)	(0.22)
Age	0.01**	− 0.02*	− 0.03**
	(0.00)	(0.01)	(0.01)
Health limits work	0.04	− 0.42	0.32
	(0.17)	(0.32)	(0.39)
Social capital	0.06	− 0.03	− 0.01
	(0.07)	(0.11)	(0.15)
Risk tolerance	− 0.03	0.04	− 0.03
	(0.02)	(0.04)	(0.06)
Retail	− 0.19	0.49†	1.10***
	(0.14)	(0.29)	(0.34)
Construction	− 0.22	0.14	− 0.18
	(0.14)	(0.22)	(0.27)
Education	0.20	Omitted	− 0.22
	(0.22)		(0.52)
Health and social work	− 0.08	0.35	0.43
	(0.14)	(0.25)	(0.46)
Recreational	0.00	− 0.89*	1.36*
	(0.20)	(0.39)	(0.61)
Full-time venture	0.08	1.24***	1.92
	(0.11)	(0.18)	(0.23)
Switch from employee	Omitted	Omitted	Omitted
Number of rooms	0.05*	− 0.06	0.10*
	(0.02)	(0.04)	(0.05)
Detached	− 0.26	0.60	− 4.53***
	(0.27)	(0.48)	(0.41)
Semi-detached	− 0.41	0.89†	− 4.41***
	(0.28)	(0.49)	(0.40)
Terraced	− 0.42	1.11*	− 3.77***
	(0.28)	(0.50)	(0.42)
Flat	− 0.41	1.11*	− 3.16***
	(0.32)	(0.55)	(0.43)
Homeowner	− 0.16	0.62*	0.97**
	(0.16)	(0.29)	(0.33)
Local house price	0.12	− 1.27**	− 1.60**
	(0.25)	(0.45)	(0.55)
Local earnings	− 0.47	1.33†	1.51*
	(0.37)	(0.71)	(0.77)
Unemployment	0.00	0.06	− 0.02
	(0.03)	(0.05)	(0.06)
Urban	− 0.05	0.01	0.11
	(0.11)	(0.21)	(0.25)
Number of observation	1588	903	725
Log pseudolikelihood	− 525.90	− 162.93	− 97.29
Wald *χ*^2^	817.02	276.74	1571.00

†*p* < 0.10

*p < 0.05

**p < 0.01

***p < 0.001 (two-tailed test)

This section closes with several robustness checks. First, we tested a probit model using stabilized weights to account for possible selection bias associated with the self-employed differing from other workers based on a set of observables. To this end, we calculated inverse probability of treatment weights (IPTW) to control for selection on observables (Azoulay et al. 2009; Fewell et al. 2004). We defined self-employed individuals as the treated group and employed individuals as the control group and computed stabilized weights for the members of both groups. Finally, we re-ran the pooled probit regression model using stabilized weights. We repeated this procedure for total sample, male subsample, and female subsample.

Table 6 displays the estimates of the pooled probit model using stabilized weights. Model (7) presents findings for the total sample, while models (8) and (9) present the findings for the male and female subsamples, respectively. Hypothesis 1, which predicted a negative relationship between being an employer and homeworking, continues to receive support—for all three samples (*p* < 0.001). Likewise, Hypothesis 2a, which predicted a negative relationship between business partnerships and homeworking, continues to receive support for male subsample (*p* < 0.05)—as does the weaker relationship for females (Hypothesis 2b). However, we discern no significant relationship between caregiving responsibilities of disabled family member and dependent child and propensity to choose homeworking, for all three samples. Therefore, Hypotheses 3a and 3b are not supported in our robustness checks. Also, once we control for sample selection, being a homeowner is *negatively* related to homeworking overall and for the female self-employed subsample. Perhaps female homeowners are wealthier than female non-homeowners and wealthier females can afford office rent for their own business, which is related to their decision to eschew homeworking. For the female subsample, the number of rooms in house is positively related to homeworking, which seems intuitive.

**Table 6:** Robustness checks: inverse probability of treatment weights

	Probit estimation, using stabilized weights		
DV = homeworker	(7) Total sample	(8) Male sample	(9) Female sample
Employer	− 0.89***	− 0.83***	− 1.25***
	(0.13)	(0.13)	(0.30)
Business partnership	− 0.19	− 0.31*	− 0.01
	(0.13)	(0.15)	(0.29)
Caregiver (disabled)	0.15	0.31	− 0.61
	(0.33)	(0.36)	(0.61)
Caregiver (child)	0.43†	− 0.11	0.43
	(0.22)	(0.52)	(0.28)
Female	− 0.04	Omitted	Omitted
	(0.20)		
Hi-tech occupation	0.34*	0.21	0.12
	(0.17)	(0.18)	(0.54)
Married	− 0.12	− 0.35	0.10
	(0.23)	(0.28)	(0.39)
Graduate	− 0.27	− 0.06	− 0.43
	(0.18)	(0.22)	(0.33)
Age	0.03***	0.03***	0.04*
	(0.01)	(0.01)	(0.01)
Health limits work	0.11	0.06	0.25
	(0.17)	(0.18)	(0.42)
Social capital	0.11	0.11	0.09
	(0.08)	(0.10)	(0.15)
Risk tolerance	0.03	0.04	− 0.02
	(0.03)	(0.03)	(0.06)
Freelance	− 0.12	0.09	− 0.78*
	(0.22)	(0.27)	(0.36)
Retail	− 0.42†	− 0.47†	− 0.41*
	(0.22)	(0.25)	(0.43)
Construction	− 0.17	− 0.16	0.90
	(0.15)	(0.16)	(0.76)
Education	0.38	0.47	0.05
	(0.25)	(0.32)	(0.44)
Health and social work	0.37	− 0.24	0.63*
	(0.24)	(0.39)	(0.32)
Recreational	0.03	0.01	− 0.45
	(0.28)	(0.32)	(0.51)
Full-time venture	0.05	0.12	− 0.10
	(0.11)	(0.12)	(0.23)
Switch	− 0.04	0.03	− 0.33
	(0.22)	(0.26)	(0.40)
Number of rooms	0.06†	0.01	0.25***
	(0.03)	(0.04)	(0.07)
Detached	− 0.48	− 0.53	− 0.57
	(0.31)	(0.44)	(0.41)
Semi-detached	− 0.57†	− 0.76†	− 0.28
	(0.31)	(0.44)	(0.43)
Terraced	− 0.46	− 0.66	0.07
	(0.33)	(0.45)	(0.48)
Flat	− 0.61†	− 0.71	− 0.27
	(0.36)	(0.49)	(0.55)
Homeowner	− 0.43*	− 0.32	− 0.94**
	(0.18)	(0.23)	(0.31)
Local house price	− 0.06	− 0.02	− 0.30
	(0.30)	(0.34)	(0.62)
Local earnings	− 0.38	− 0.47	0.26
	(0.44)	(0.54)	(0.84)
Local unemployment	0.01	0.02	0.03
	(0.03)	(0.03)	(0.06)
Urban	− 0.14	− 0.12	− 0.41†
	(0.12)	(0.14)	(0.24)
Number of observation	1964	1439	525
Log pseudolikelihood	− 1090.49	− 782.23	− 273.12
Pseudo *R*^2^	0.17	0.18	0.24
Wald *χ*^2^	152.97	145.93	87.58

†*p* < 0.10

*p < 0.05

**p < 0.01

***p < 0.001 (two-tailed test)

Second, we tested whether interaction variables of different items related to caregiving responsibilities are related to the homeworking decision of entrepreneurs. In additional (unreported) tests, we found that the interaction of providing caregiving to a disabled person and providing caregiving to a dependent child was not a significant predictor of self-employed homeworking. Also, the interaction of providing caregiving to a disabled person and providing caregiving to a dependent child as a single parent was not significantly related to self-employed homeworking, either. Nor was the interaction of providing caregiving with single parenthood.

Third, the number of hours worked in a business may affect the propensity to choose homeworking. We did not include this variable in the full model earlier because of the possibility it might be endogenous. Yet adding this variable to the model yielded an insignificant coefficient (details available on request). Fourth, we tested whether age has different functional forms such as quadratic or discontinuities around 65. However, we did not find significant effects from including higher-order terms or age discontinuity dummies (results also available on request). We therefore conclude that our results appear to be fairly robust to different model specifications.

## Conclusion

This article has highlighted an important but hitherto largely overlooked aspect of entrepreneurship, namely the prevalence of homeworking among the self-employed. Nearly two-fifths of the UK self-employed are homeworkers; yet, previous research has paid surprisingly little attention to such a populous group of entrepreneurs. To fill this gap, we proposed some novel hypotheses about the determinants of entrepreneurial homeworking and tested them using a large sample of panel data.

The most notable finding is that homeworking does not seem to serve as a transitional state for fledgling new businesses before they scale and professionalize and transition to non-homeworking firms. If it did, one might expect to observe numerous “hybrid” entrepreneurs, who work primarily in paid employment practicing self-employment as a second job, transitioning into full-time non-homeworking self-employment. We would also expect to observe entrepreneurs who take on employees and business partners to transition away from homeworking and into non-home-based businesses. In fact, the evidence failed to support any of these predictions. One is therefore led to conclude that homeworking is an integral and stable manifestation of entrepreneurship.

That being the case, we went on to explore what factors influence individuals’ decisions to choose homeworking. We found that self-employed homeworkers are significantly less likely to be employers or to have business partners, with stronger relationships between these variables observed for male than female entrepreneurs. We found a significant relationship between providing caregiving to a dependent child and homeworking among the self-employed, for women specifically. However, to our surprise, we detected no significant relationship between providing caregiving to disabled family members and self-employed homeworking among our respondents, including for the female sample.

The lack of a significant association between caregiving of disabled family members and self-employed homeworking seems to fly in the face of prior theorizing and previous empirical findings. One possibility is that self-employment provides enough flexibility in the British context to enable entrepreneurs to discharge their caregiving obligations to disabled family members, such that homeworking confers no additional advantages in this regard. The precise nature of the ventures that the self-employed operate may be relevant in this regard: exploring this possibility further and in greater detail provides an opportunity for future research.

Our findings may carry implications for entrepreneurship scholars wrestling with two puzzles. One puzzle is the limited number of entrepreneurs who create jobs for others—an important issue given strong policy interest in, and encouragement, of job creation (Caliendo and Kritikos 2010; Henley 2005; Mathur 2010; Michaelides and Benus 2012). The present article has identified homeworking as a hitherto overlooked explanation for limited job creation. Homeworking can help explain the limited scale of most entrepreneurial ventures (Davidsson et al. 2009; Hurst and Pugsley 2011) and their surprisingly limited growth ambitions (Delmar and Wiklund 2008; Wiklund and Shepherd 2003). The logistical difficulties of operating a multi-person venture from a domestic property, together with the agency costs entailed by spatially separating employees from the entrepreneur owner-manager, could account for incompatibility between entrepreneurial homeworking and job creation. Combined with the prevalence and persistence of homeworking, the weak job creation performance of the self-employed may become more understandable.

A second puzzle in the entrepreneurship literature is the apparent earnings penalty entailed by self-employment; contrary to the earnings premium, one might expect the self-employed to receive in return for bearing greater risk (Hall and Woodward 2010; Manso 2016). If self-employed homeworkers earn less on average than their non-homeworker counterparts, e.g., because of a positive “compensating differential” derived from working at home, then this might explain part of the puzzle. The idea behind the compensating differential is that a pleasant non-financial feature of a working arrangement (e.g., being based at home) can compensate for lower financial remuneration, thereby inducing some entrepreneurs to take low-income business opportunities which they might otherwise forgo.

Consistent with this logic, some income calculations by the authors using the BHPS revealed a much greater pre-tax earnings penalty for homeworker self-employed than for non-homeworker self-employed. These calculations used data on pre-tax earnings, and computed averages for paid employees, homeworker self-employed, and non-homeworker self-employed. They revealed that the average pre-tax earnings penalty of all self-employed relative to employees was £240 per month, whereas self-employed homeworkers earned on average £738 per month less than the non-homeworker self-employed (all amounts in 2008 prices). In fact, were one to remove homeworkers from the self-employed sample, the £240 per month self-employment penalty relative to employees would be turned into a £58 per month *premium*. Hence, homeworking may partly explain lower average overall self-employment incomes compared with employees.

Homeworking is not only widespread but also carries implications for policymakers wanting to promote entrepreneurship. To start with, growing evidence suggests that locally based entrepreneurship contributes significantly to the development of peripheral regions (Baumgartner et al. 2013; Stephens and Partridge 2011). In the USA, for example, home-based ventures make an especially important economic contribution in places that struggle to attract large retailers or manufacturing plants (Rowe et al. 1999). Entrepreneurial homeworking might therefore be crucial for revitalizing local communities facing geographic and economic barriers.

We generated some tentative evidence (Table 6, column (7)) that high-tech workers are more likely to become self-employed homeworkers. To the extent that high-tech workers need access to high-quality digital infrastructure, our research might also carry implications for technology policy. For example, Malecki (2003) has highlighted shortcomings in the US digital infrastructure which may be holding peripherally located businesses back. In the UK, in March 2020, Chancellor of the Exchequer Rishi Sunak promised more than £5bn of investment into Britain’s digital infrastructure, to be used to spread gigabit broadband to remote corners of the UK, benefitting more than five million homes and businesses. This Budget announcement followed a pledge by Prime Minister Johnson that every home would have access to the next generation of Internet technology by 2025. This type of infrastructure investment, which is rolling out in many other countries as well, is likely to increase the ease with which tech-based businesses can be operated at home, especially given continued growth in the number of Internet-based ventures (and possibly also in response to the Covid-19 pandemic). Given ongoing discussions about “the future of work” which may involve greater use of homeworking and self-employment, this initiative and others like it could fuel growth in the number of entrepreneurial homeworkers in the years ahead.

Further research is needed to quantify the social welfare implications of entrepreneurial homeworking relative to alternative work arrangements. That could shed light on the impact of other policy instruments such as zoning laws and rent subsidies, which might also affect the entrepreneurial choices explored in this article. Mason et al. (2011) noted that planning laws, regulations, and tax rules were formulated decades or even centuries before the digital knowledge-based economy was envisaged. Policy changes may be needed to support home-based entrepreneurship in this new context, for example by allowing social housing residents to start home-based businesses to escape poverty and worklessness. The task of formulating detailed policies in this regard is likely to be complex and multi-faceted: we leave it to future work.

Future research may also seek to address several limitations of the present article. First, the present paper abstracted from several important considerations, including endogenous worker mobility, entrepreneur mobility, and the determination of business office rents. Some prior research (e.g., Rosenthal and Strange 2012) has explored some of these issues, though not in the context of homeworking decisions. A fuller treatment of these issues would seem worthwhile. Second, among the empirical limitations of this article one may question the use of self-employment as a measure of entrepreneurship. It would be interesting to know whether the results obtained here continue to hold for alternative definitions of entrepreneurship; and to quantify the amount, type, and determinants of homeworking using alternative definitions. Third, it would be valuable to utilize data from other countries and investigate temporal variations and trends in the phenomenon of self-employed homeworking. These tasks are all left for future research.

Fourth, industry controls and possibly several other variables, such as work hours and earnings, may be jointly determined with homeworking. Thus, the paper should be regarded as exploratory and correlational in nature: it does not make strong claims about uncovering causal relationships. Fifth, our investigation did not exhaust all forms of risk-minimization strategies associated with homeworking. For instance, it is possible that some self-employed homeworkers organize as sole proprietors, and switch to incorporated status and non-homeworking. We lack the data to explore this possibility in our study, though we note that prior research suggests that this kind of transition is infrequent (Åstebro and Tåg 2017).

To conclude, entrepreneurial homeworking is simultaneously a widespread and a sorely understudied phenomenon. More research is needed on this topic, in part to inform entrepreneurs and policymakers, and in part to build out scholarly understanding of entrepreneurship. Its prevalence alone makes it deserve of further attention; but one can also imagine a whole set of questions which researchers can explore anew by distinguishing between homeworker and non-homeworker entrepreneurs. For example, are borrowing constraints different for the two groups of entrepreneurs, and are the growth and survival prospects of their ventures different too? How does the growing trend of flexible “gig economy” working and multiple job holding (Adams et al. 2018) intersect with homeworking among the self-employed, and what are the likely directions this may take in the future? What are the regional impacts of homeworking in terms of economic development and living standards? These are all interesting questions which the homeworker distinction open up for fresh investigation, and which promise to generate further unexpected and fruitful insights.

## Appendix 1

**Table 7:** High-tech occupations

Code	Occupation
2111-2132	Science and technology professionals
2321-2329	Research professionals
2411-2434	Legal professionals; business and statistical professionals; architects, town planners, surveyors
3111-3132	Science and technology associate professionals
3421-3434	Design associate and media associate professionals
3520-3539	Legal and business and finance associate professionals

**Table 8:** Retail industry sectors

Code	Description	Number of homeworking cases
5000	Sale, maintenance, and repair of motor vehicles and motorcycles; retail sale of automotive fuel	12
5100	Wholesale trade and commission trade, exc. motor vehicles and motorcycles	42
5200	Retail trade, exc. motor vehicles and motorcycles; repair of personal and household goods	3
5210	Retail sale in non-specialized stores	7
5220	Retail sale of food, beverages, and tobacco in specialized stores	10
5240	Other retail sale of new goods in specialized stores	13
5250	Retail sale of second-hand goods in stores	2

**Table 9:** Construction industry sectors

Code	Description	Number of homeworking cases
4500	Construction	0
4510	Site preparation	0
4520	Building of complete constructions or parts thereof; civil engineering	70
4530	Building installation	60
4540	Building completion	78
4550	Renting of construction or demolition equipment with operator	4

**Table 10:** Education industry sectors

Code	Description	Number of homeworking cases
8000	Education	1
8010	Primary education	0
8020	Secondary education	21
8030	Higher education	10
8040	Adult and other education	68

**Table 11:** Health and social work industry sectors

Code	Description	Number of homeworking cases
8500	Health and social work	0
8510	Human health activities	28
8520	Veterinary activities	0
8530	Social work, community, and counseling activities	109

**Table 12:** Recreational, cultural, and sporting activities industry sectors

Code	Description	Number of homeworking cases
9200	Recreational, cultural and sporting activities	0
9210	Motion picture and video activities	1
9220	Radio and television activities	1
9230	Other entertainment activities	64
9240	News agency activities	20
9250	Library, archives, museums, and other cultural activities	3
9260	Sporting activities	5
9270	Other recreational activities	3

## Appendix 2. Data Sources for LAD level variables

1. House Price Index

UK House Price Index, http://landregistry.data.gov.uk/app/ukhpi

2) House sales data

Office for National Statistics (ONS), earnings and hours worked, place of residence by local authority: ASHE Table 8https://www.ons.gov.uk/employmentandlabourmarket/peopleinwork/earningsandworkinghours/datasets/placeofresidencebylocalauthorityashetable8

3) ASHE LAD lookup file

Lookup file received by email from earnings@ons.gov.uk

4) Local unemployment rate

Office for National Statistics (ONS), Official labor market statistics

https://www.nomisweb.co.uk/query/construct/summary.asp?mode=construct&version=0&dataset=17

5) Urban/rural classifications

England: Government of UK, 2011 Rural-Urban Classification of Local Authorities and other geographies, https://www.gov.uk/government/statistics/2011-rural-urban-classification-of-local-authority-and-other-higher-level-geographies-for-statistical-purposes

Scotland: Scottish Government, Scottish Government Urban Rural Classification 2007–2008, https://www.gov.scot/publications/scottish-government-urban-rural-classification-2007-2008/pages/4/

Wales: Rural Urban Classification (2011) of Middle Layer Super Output Areas in England and Wales, http://geoportal.statistics.gov.uk/datasets/rural-urban-classification-2011-of-middle-layer-super-output-areas-in-england-and-wales
